# Inhibition of the CDK4/6-Cyclin D-Rb Pathway by Ribociclib Augments Chemotherapy and Immunotherapy in Renal Cell Carcinoma

**DOI:** 10.1155/2020/9525207

**Published:** 2020-06-11

**Authors:** Dehong Chen, Xiaosong Sun, Xuejun Zhang, Jun Cao

**Affiliations:** Department of Urology, Xiangyang Central Hospital, Affiliated Hospital of Hubei University of Arts and Science, Xiangyang, Hubei, China 441021

## Abstract

Renal cell carcinoma (RCC) is the most aggressive type of genitourinary cancer and is resistant to current therapies. Identifying drugs that enhance the efficacy of RCC standard-of-care drugs at sublethal concentrations is an alternative therapeutic strategy. Ribociclib is an orally available cyclin-dependent kinase 4 and 6 (CDK4/6) inhibitor that is approved for the treatment of breast cancer. In this work, we demonstrate that ribociclib at clinically achievable concentrations inhibits proliferation of 7 out of 9 tested RCC cell lines, with IC_50_ range from 76 to 280 nM. In addition, ribociclib induces apoptosis of RCC cells, but with less potency compared to its antiproliferative activity. The combination of ribociclib with chemotherapeutic or immunotherapeutic agents is synergistic in RCC cell lines. Of note, ribociclib demonstrates selective anti-RCC activity by sparing normal kidney cells and fibroblast cells. Consistent with the *in vitro* findings, ribociclib inhibits RCC growth at the dosage that does not lead to toxicity in mice and enhances the *in vivo* efficacy of RCC standard-of-care drugs. Mechanistically, we show that ribociclib remarkably inhibits phosphorylation of retinoblastoma protein (Rb) at various sites, leading to the suppression of transcription of E2F target genes in RCC cells. Our findings clearly demonstrate the potency and selectivity of ribociclib in RCC preclinical models, via inhibition of the CDK4/6-cyclin D/Rb pathway. Our findings support a clinical trial for the combination of ribociclib with chemo/immunotherapy in RCC.

## 1. Introduction

Renal cell carcinoma (RCC) accounts for 90% of kidney tumors with poor prognosis, and its incidence is increasing over the past decades [1]. RCC is a clinicopathologically heterogenous disease that can be classified into clear cell carcinoma, papillary carcinoma, chromophobe carcinoma, collecting duct carcinoma, and medullary carcinoma subtypes [2]. 35% of RCC patients are diagnosed at the metastatic stage with median survival time of less than 18 months [3]. Systemic therapy including chemotherapy (e.g., fluorouracil (5-FU)), immunotherapy (e.g., interferon (IFN-*α*)), and targeted therapy (e.g., sorafenib) has not significantly improved the clinical outcome in RCC [4]. Identifying drugs that can sensitize RCC to standard-of-care drugs while sparing normal cells is needed for RCC treatment.

The retinoblastoma (RB) tumor suppressor is a known master regulator of the cell cycle that slows cell division by preventing the progression of the cell cycle from the G1 to S phase [5]. The phosphorylation of Rb leads to Rb inactivation and induces cell cycle deregulation [6]. Cancer cells overcome the Rb-dependent restriction point through alterations that lead to constitutive activation of cyclin D-cyclin-dependent kinase 4/6 (CDK4/6) [6]. The CDK4/6-cyclin D-Rb pathway has been identified as a rational target for cancer therapy [7]. Increased expression of CDKs and cyclin D1 has been observed in RCC [8–10]. CDK1 and CDK2 activity is a strong predictor of renal cell carcinoma recurrence [9]. Loss of CDK4/6 activity is synthetically lethal with VHL inactivation in clear cell RCC [11]. Ribociclib is a potent and selective CDK4/6 dual inhibitor leading to the activation of Rb and cell cycle arrest [12]. It is an FDA-approved drug for the treatment of hormone receptor-positive, human epidermal growth factor receptor 2-negative metastatic breast cancer in combination with endocrine agents [13]. In addition, preclinical findings demonstrate that ribociclib displays preferentially anticancer activities in liposarcoma and neuroblastoma [14, 15] .

In this work, we investigated the *in vitro* and *in vivo* efficacy of ribociclib alone and its combination with RCC standard-of-care drugs. In addition, we attempted to identify the mechanism of action of ribociclib in RCC cells focusing on Rb signaling.

## 2. Materials and Methods

### 2.1. Cells and Drug Treatment

Seven human RCC cell lines (786-O, CaKi-1, Caki-2, A-704, 769-P, A498, and ACHN), three human immortalized normal kidney cell lines (HEK-293, RPTEC/TERT1, and CCD1103), and a normal human fibroblast cell line (BJ) were obtained from ATCC. Two human RCC cell lines SW839 and UM-RC-2 were obtained from the Cell Bank of Type Culture Collection of the Chinese Academy of Sciences. All cell lines were maintained in the Key Laboratory of Hubei University of Arts and Science. Cells were cultured in Eagle's Minimal Essential Media (MEM) supplemented with 10% fetal bovine serum (HyClone, UK), 1% HEPES (Life Technologies, USA), and penicillin/streptomycin in a 37°C atmosphere with 5% CO_2_ and 20% O_2_. Interferon-*α* (IFN-*α*, SRP4595, Sigma) was reconstituted in water. Ribociclib (HY-15777, MCE) and 5-Fluorouracil (5-FU, F6627, Sigma) were reconstituted in DMSO. For single drug treatment, ribociclib at concentrations ranging from 0.05 to 0.8 *μ*M was added to the well. For combination studies, ribociclib, 5-FU, and IFN-*α* alone at one single dose, the combination of ribociclib with 5-FU, and the combination of ribociclib with IFN-*α* were added to the well.

### 2.2. Measurement of Proliferation

5 × 10^3^ cells/well were seeded to a 96-well plate. The next day, drugs were added to the well and incubated for 72 hours. Cell proliferation activity was assessed using the Bromodeoxyuridine (BrdU) Cell Proliferation Assay Kit as per the manufacturer's protocol.

### 2.3. Measurement of Apoptosis

5 × 10^5^ cells/well in a 12-well plate were seeded. The next day, drugs were added to the well and incubated for 72 hours. The treated cells were trypsinized and resuspended in PBS. Cells were stained using the Annexin V-FITC/7-AAD (BD Pharmingen, USA) Kit as per the manufacturer's protocol. The stained cells were analysed on Beckman Coulter FC500 with a minimum of 10,000 events counted. Annexin V+/7-AAD- and Annexin V+/7-AAD+ cells were considered apoptotic cells.

### 2.4. Western Blot Analyses

5 × 10^6^ cells/well in a 6-well plate were seeded. The next day, drugs were added to the well and incubated for 24 hours. The treated cells were lysed at 4°C in radioimmunoprecipitation assay (RIPA) buffer (Invitrogen, USA). Insoluble materials were cleared by centrifugation, and the supernatant was collected for protein concentration measurement using a BCA protein assay kit (Thermo Scientific, USA). An equal amount of proteins was resolved by SDS-PAGE and was transferred to a PVDF membrane. Total Rb, phosphor Rb, and p16INK4a were detected using antibodies purchased from Santa Cruz Biotechnology, Inc.

### 2.5. RT-PCR

5 × 10^6^ cells/well in a 6-well plate were seeded. The next day, drugs were added to the well and incubated for 24 hours. Total RNA in the treated cells was isolated using TRIzol (Invitrogen, USA). RT-PCR was performed using the Superscript One-Step RT-PCR kit (Invitrogen, USA) as per the manufacturer's protocol. Primer sequences are as follows: FOXM1—forward: 5′-GGT GTG AAT GAA GAC TTG GCT GA-3′ and reverse: 5′-GTT TCA TCC AGG ATG GCT TGG CA-3′, CCNE1—forward: 5′-ACG AAG GTC TGC GCG TGT T-3′ and reverse: 5′-CCG CTG GCC ATG AAC TAC CT-3′, and CDC6—forward: 5′-TGT CAA AAG CCA GAC TAT-3′ and reverse: 5′-GTG AAT AAG ACC AAC CCT-3′.

### 2.6. RCC Tumor Xenograft in SCID Mice

The animal experiments conformed to the guidelines set forth by the Institutional Animal Care and Use Committee of Xiangyang Central Hospital. RCC xenografts were generated by subcutaneous injection of 1 million 786-O or CaKi-1 cells into the flank of 6-week-old male NOD/SCID mice. Tumor size and body weight were monitored every alternative day. After the development of palpable tumors (~200 mm^3^), mice were randomized into the following six groups (*n* = 10 per group): (i) control arm receiving 20%/80% DMSO/saline, (ii) intraperitoneal 5-FU at 1 mg/kg once every three days, (iii) intraperitoneal 10^5^ units of pegylated IFN-*α* (Schering-Plough, US) once every 3 days, (iv) oral ribociclib at 50 mg/kg daily, (v) combination of ribociclib and 5-FU, and (vi) combination of ribociclib with IFN-*α*. The dosing interval was determined according to published literatures as well as our preliminary results. The dose of a drug that leads to slight or moderate inhibition of tumor growth (<40%) as a single drug alone was chosen for combination studies. All mice were euthanized at the end of the 3-week treatment.

### 2.7. Statistical Analyses

Data are expressed as mean and standard deviation. Statistical analyses are performed by one-way analysis of variance followed by Tukey's HSD test for multiple comparisons or unpaired Student's *t*-test for pairwise comparisons. *p* < 0.05 is considered statistically significant.

## 3. Results

### 3.1. Ribociclib Is Selective in Targeting RCC Cells

**Figure 1:**
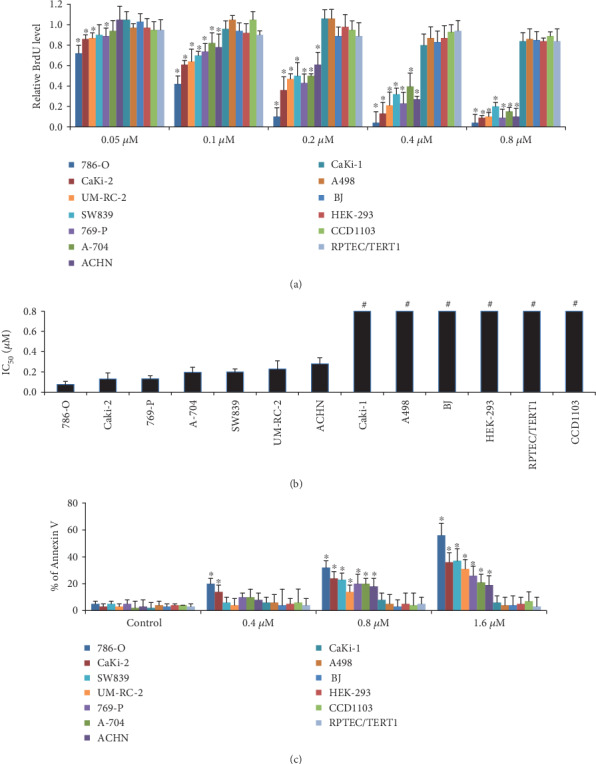
The sensitivity of RCC cancer cell lines and normal cells to ribociclib. (a) The differential antiproliferative effects of ribociclib in multiple RCC cell lines and normal cells. BJ is fibroblast cells. HEK-293, RPTEC/TERT1, and CCD1103 are normal kidney cell lines. 786-O, SW839, CaKi-2, CaKi-1, and A-498 are clear cell RCC cell lines. 769-P, A-704, UM-RC-2, and ACHN are renal adenocarcinoma cell lines. Results shown are relative to control (value set as 1). (b) IC_50_ values of ribociclib in RCC and normal cells. Cell lines are ordered from left to right with low to high IC_50_. # indicates that the IC_50_ value is more than the highest dose tested. (c) The differential proapoptotic effects (shown by % of Annexin V) of ribociclib in multiple RCC cell lines and normal cells. Cells were treated with the indicated doses for 72 h prior to proliferation and apoptosis analysis. ^∗^*p* < 0.05, compared to control (value is 1).

The inhibitory effects of ribociclib and its 50% inhibitory concentration (IC_50_) for a panel of RCC and normal cell lines were evaluated using a range of concentrations from 0.0125 to 1.6 *μ*M. All the concentrations tested in our study are clinically achievable as the plasma concentration of ribociclib up to 6 *μ*g/ml was detected in patients receiving ribociclib therapy [16]. Nine human RCC cell lines selected in our study cover different histological types with varying genetic profiling [17]. Ribociclib potently inhibited the proliferation in 7 out of 9 tested RCC cell lines in a wide concentration-dependent manner (Figure 1(a) and Supplementary Figs. S1 and S2). The IC_50_ range of ribociclib in these 7 RCC cell lines was from 76 to 280 nM (Figure 1(b) and Supplementary Table 1). Ribociclib up to 0.8 *μ*M did not inhibit proliferation in 2 out of 9 RCC cell lines and normal cell lines including normal kidney cells (HEK-293, RPTEC/TERT1, and CCD1103) and fibroblast cells (BJ) (Figure 1(a)). The IC_50_ value of these cell lines was not reached within the range of concentrations (0.0125 to 0.8 *μ*M) tested (Figure 1(b)). We further found that ribociclib up to 1.6 *μ*M induced 19% to 56% apoptosis in 7 out of 9 RCC cell lines (Figure 1(c) and Supplementary Fig. 3). Ribociclib did not induce apoptosis in normal cells (Figure 1(c)). Based on the findings obtained from proliferation and apoptosis assays, CaKi-1 and A498 cells are resistant to ribociclib.

### 3.2. Ribociclib Enhances the In Vitro Efficacy of a Chemotherapeutic or Immunotherapeutic Agent in Some but Not All Tested RCC Cell Lines

**Figure 2:**
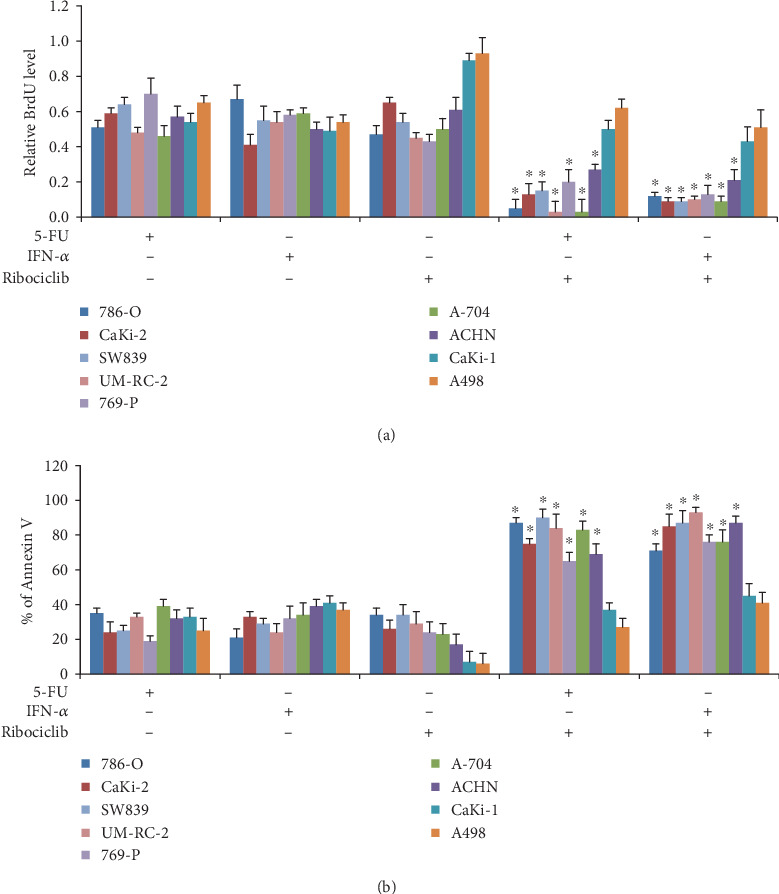
Ribociclib significantly enhances the *in vitro* efficacy of chemotherapy and immunotherapy in sensitive but not resistant RCC cell lines. Combination of ribociclib with 5-FU (chemotherapeutic agent) or IFN-*α* (immunotherapeutic agent) is significantly more effective in inhibiting proliferation (a) and inducing apoptosis (b) than 5-FU or IFN-*α* alone in sensitive but not resistant cell lines. Results shown in (a) are relative to control (value set as 1). 5-FU at 1 *μ*M and IFN-*α* at 100 IU/ml were used in the combination assays in all tested RCC cell lines. Ribociclib at 0.1 *μ*M in 786-O, CaKi-2, SW839, and UM-RC-2 cell lines, at 0.2 *μ*M in 769-P, A-704, and ACHN, and at 0.8 *μ*M in CaKi-1 and A498 cell lines was used for the proliferation assay. Ribociclib at 0.8 *μ*M in 786-O, CaKi-2, SW839, and UM-RC-2 cell lines and at 1.6 *μ*M in 769-P, A-704, ACHN, CaKi-1, and A498 cell lines was used for the apoptosis assay. ^∗^*p* < 0.05, compared to 5-FU or IFN-*α* alone.

To correlate with clinical trials that test the efficacy of ribociclib in the addition of other anticancer agents (ClinicalTrials.gov Identifier: NCT03078751 and NCT03822468), we determined the combinatory effects of ribociclib with RCC standard-of-care drugs: the immunotherapeutic agent IFN-*α* and the chemotherapeutic agent 5-FU [18]. In order to achieve the linear range of combinatory effects, drugs used in combination studies were tested at a concentration that results in <40% inhibition as a single drug alone. As shown in Figure 2, ribociclib significantly augmented the antiproliferative and proapoptotic effects of both IFN-*α* and 5-FU in those RCC cell lines that are sensitive to ribociclib as a single drug alone. In addition, ribociclib did not enhance the efficacy of IFN-*α* and 5-FU in those RCC cell lines that are resistant to ribociclib. These results suggest that the combination achieves greater efficacy than the single drug alone only in RCC cell lines that ribociclib is active against.

To investigate whether the combination of ribociclib with 5-FU or IFN-*α* is synergistic, we performed combination studies using the Chou-Talalay method [19] and calculated the combination index (CI) using the CalcuSyn software. The IC_50_ of the drug alone or in combination is shown in Supplementary Table 1. CI20, CI50, and CI80 were all less than 1 in 786-O, CaKi-2, 769-P, A704, ACHN, SW839, and UM-RC-2 cells (Tables 1 and 2). In contrast, CI values were all more than 1 in CaKi-1 and A498 cells (Tables 1 and 2). These results indicate that the combination of ribociclib with 5-FU or IFN-*α* is synergistic in ribociclib-sensitive but not ribociclib-resistant RCC cells.

### 3.3. Ribociclib Acts on RCC via Inhibiting CDK4/6-Cyclin D-pRb Signaling

**Figure 3:**
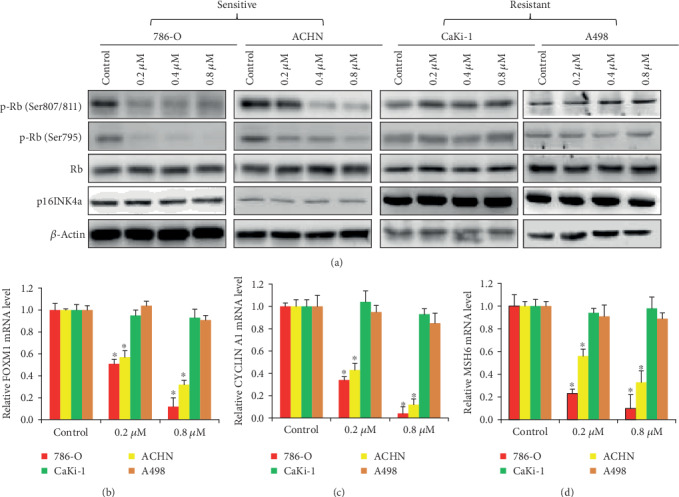
Differential effects of ribociclib on the CDK4/6-cyclin D-pRb signaling in RCC. (a) The representative western blot photo shows decreased and unchanged level of p-Rb at two phosphorylation sites in sensitive (IC_50_ < 200 nM) and resistant (IC_50_ > 800 nM) RCC cell lines, respectively. (b) The decreased and unchanged transcriptional level of E2F target genes: FOXM1, CCNE1, and MSH6 in RCC cell lines. Cells were treated with ribociclib for 24 hours prior to western blot and mRNA level analysis. ^∗^*p* < 0.05, compared to control.

Constitutive phosphorylation and inactivation of Rb function by CDK4/6 partners with D-type cyclins [7]. To investigate whether the action of ribociclib in RCC cells is associated with its ability to inhibit the CDK4/6-cyclin D-Rb pathway, we firstly determined the level of Rb phosphorylation in multiple RCC cell lines that are both sensitive and resistant to ribociclib. We found that ribociclib repressed RB phosphorylation at Ser807/811 and Ser795 but not the total Rb level in 786-O and ACHN cells, and pRB levels were dose-dependently reduced with the treatment with ribociclib (Figure 3(a)). In contrast, ribociclib did not affect RB phosphorylation in CaKi-2 and A498 cells. p16INK4a (CDKN2A) is a critical component of the CDK4/6 complex, and its high protein expression in cancer cells often indicates Rb loss of function [20]. We found that ribociclib treatment did not affect p16INK4a level in RCC cells regardless of their sensitivity to ribociclib (Figure 3(a)). We further found that ribociclib-resistant lines A498 and CaKi-2 displayed higher baseline expression of p16INK4a than ribociclib-sensitive lines (Supplementary Fig. 4). This is consistent with the previous reports that sensitivity of RCC cells to CDK4/6 inhibitors is associated with many factors, such as expression of p16 [21].

Phosphorylated Rb binds to members of E2F family transcription factors, which control the expression of genes that support cell cycle progression [22]. We found that the transcriptional level of FOXM1, CCNE1, and MSH6, which are E2F targeted genes [23], was remarkably decreased in 786-O and ACHN but not CaKi-1 or A498 cells exposed to ribociclib (Figure 3(b)). Consistent with their RNA levels, western blot analysis showed the decreased protein level of FOXM1, CCNE1, and MSH6 in 786-O and ACHN but not CaKi-1 or A498 cells after ribociclib treatment (Supplementary Fig. 5). The inhibitory effects of ribociclib on the Rb pathway observed only in sensitive but not resistant RCC cells clearly indicate that ribociclib acts on RCC cells through inhibiting the CDK4/6-cyclin D-pRb signaling.

### 3.4. Ribociclib Enhances the In Vivo Efficacy of a Chemotherapeutic or Immunotherapeutic Agent in Some but Not All Tested RCC Xenograft Mouse Models

**Figure 4:**
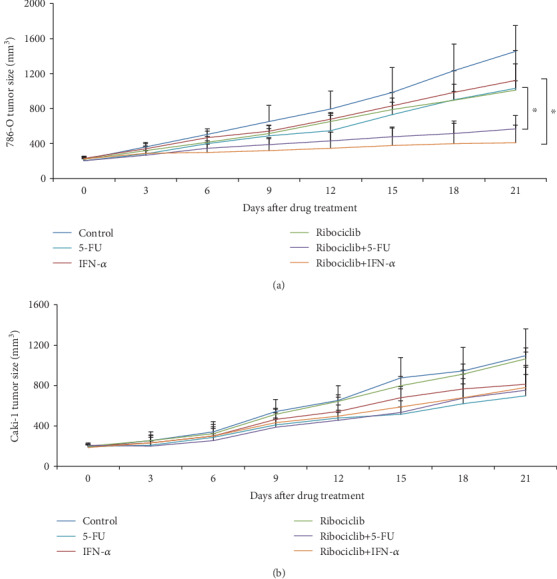
Ribociclib significantly enhances the *in vivo* efficacy of chemotherapy and immunotherapy in a sensitive but not resistant xenograft RCC model. Combination of ribociclib with 5-FU or IFN-*α* is significantly more effective in inhibiting 786-O (a) but not CaKi-1 (b) tumor growth than 5-FU or IFN-*α* alone. The RCC xenograft mouse model was established using 786-O or Caki-1 cells. ^∗^*p* < 0.05, compared to 5-FU or IFN-*α* alone.

We finally evaluated whether the combination of ribociclib with a chemotherapeutic or immunotherapeutic agent results in greater efficacy than a single agent alone in xenograft mouse models. Based on our *in vitro* data, ribociclib is active against 786-O but not CaKi-1 cells (Figures 1–3). Consistently, ribociclib at 50 mg/kg given by oral gavage mildly inhibited xenograft mouse tumor growth established by using 786-O but not CaKi-1 cells (Figure 4(a)). We observed the almost complete inhibition of 786-O tumor growth in mice receiving the combination treatment (Figure 4(a)). In addition, mice tolerated well all the treatments as we did not observe significant body weight loss throughout the whole duration of drug treatment (Supplementary Fig. 6). However, the combination of ribociclib with 5-FU or IFN-*α* did not result in greater efficacy in inhibiting CaKi-1 tumor growth (Figure 4(b)). Taken together, our results demonstrate that ribociclib enhances *in vivo* efficacy of a chemotherapeutic or immunotherapeutic agent in some but not all tested RCC xenograft mouse models.

## 4. Discussion

The landscape of therapy for RCC has rapidly evolved in the past decade from limited therapeutic options to one where includes kinase and immune checkpoint inhibitors and cytokines [24]. Contemporary research is now focusing on combinatory therapy to maximize the efficacy and minimize the toxicity. To identify drugs with complementary or synergistic effects in combination with standard-of-care drugs, we investigated the clinically existing drugs with known targets that play essential roles in RCC. A significant advantage of FDA-approved drugs over investigational compounds is the rapid clinical translation due to well-known pharmacological and toxicity profiling. Our earlier work demonstrated that the antiviral drug ribavirin cooperated with chemotherapy and immunotherapy in RCC, via inhibition of eukaryotic translation initiation factor 4E [25]. In the present study using cell culture and xenograft mouse models, we found that inhibition of the CDK4/6-cyclin D-Rb pathway by ribociclib augmented the efficacy of 5-FU and IFN-*α* and displayed selective anti-RCC activity.

We show that ribociclib is a potential candidate for RCC treatment because ribociclib at clinically achievable concentration (1) potently inhibits growth in multiple RCC cell lines (Figure 1), (2) displays selective anti-RCC activity by sparing normal cells (Figures 1(a) and 1(c)), and (3) remarkably enhances the inhibitory effects of both chemotherapeutic and immunotherapeutic agents in mice without causing toxicity (Figures 2 and 4 and Table 1). The selective anti-RCC activity of ribociclib as a single agent is consistent with the fact that the CDK4/6 pathway plays a crucial role in the proliferation and progression of cancer and serves as a promising therapeutic target in various human malignances [26–28]. The synergistic effect of ribociclib in combination with other anticancer agents in RCC is also supported by the previous work suggesting that ribociclib is a versatile combination partner in preclinical cancer models [29]. Ribociclib, palbociclib, and abemaciclib are orally administered small-molecule inhibitors of CDK4/6. In addition, ribociclib has similar potency as palbociclib in inhibiting CDK4 and CDK6 with less gastrointestinal toxicity than abemaciclib [30]. Apart from breast cancer, increasing clinical trials are currently investigating the efficacy of ribociclib in other types of cancers including lung cancer (NCT02292550), ovarian cancer (NCT03056833), and advanced neuroendocrine tumors (NCT03070301). Our findings may accelerate the initialization of clinical trials on the safety and efficacy of ribociclib and standard-of-care drugs in patients with RCC.

The principal function of cyclin D-CDK4/6 activity is to initiate the phosphorylation of Rb at sites like Ser807/811 and Ser795 that provoke functional inactivation of Rb and the subsequent release of E2F transcription factors which drive the expression of genes that support DNA synthesis and S phase progression [7]. The molecular mechanism of action of ribociclib in RCC is through inhibiting the CDK4/6-cyclin D1-Rb pathway as shown by our findings that (1) ribociclib inhibits the phosphorylation of Rb at Ser807/811 and Ser795 (Figure 3(a)), (2) ribociclib decreases the transcriptional level of E2F target genes (Figure 3(b)), and (3) ribociclib does not affect Rb phosphorylation and mRNA level of E2F target genes in RCC cells that are resistant to ribociclib (Figure 3). It is interesting to note that not all tested RCC cell lines are sensitive to ribociclib. The similar phenomenon has been reported by Finn et al. and Logan et al. that only some cell lines of breast cancer and RCC with certain subtypes and molecular features are sensitive to palbociclib [21, 31]. Consistently, the resistant RCC cell lines identified in our study such as CaKi-1 and A498 are also resistant to palbociclib. The insensitivity of CCD1103 to ribociclib is highly likely due to the expression of E6/E7 which will corrupt both p53 and Rb pathway integrities and render them unfunctional. Although the sensitivity of RCC cells to CDK4/6 inhibitors is associated with many factors, including expression of p16, p15, Rb1, and E2F1 [21], we speculate that the possible reason for the selectivity between normal and RCC cells might be that normal cells are less dependent on the CDK4/6-p16-Rb axis for proliferation and survival than RCC cells. Kim et al. shows that ribociclib is more active in CDK4-dependent cell lines than in CDK6-dependent cell lines [29]. In our study, CaKi-1 and A498 cells are resistant to ribociclib because ribociclib does not affect the phosphorylation of Rb although these cells express Rb (Figure 3(a)). This suggests that (1) Rb alone is not predictive of response to ribociclib, (2) potential mutation in CDK4/6 leads to loss of binding and kinase inhibition, and (3) other mechanisms drive Rb hyperphosphorylation, in resistant RCC cell lines.

In conclusion, our work is the first to demonstrate the selectivity and synergistic effects of ribociclib with RCC standard-of-care drugs in preclinical settings. Ribociclib has advantages over many other investigational compounds given the well-known information on drug molecular target, toxicity, and pharmacokinetics. Our findings support further investigation of ribociclib in RCC in clinical settings.

## Figures and Tables

**Table 1:** Combination of ribociclib and 5-FU is synergistic in inhibiting the proliferation of some RCC cell lines. Combination studies were performed using the Chou-Talalay method. Briefly, the IC_50_ of the single drug was determined in the single-arm experiments. Combination studies were performed by treating cells with a single drug alone and an equipotent constant-ratio (according to IC_50_) combination of two drugs. The combination index (CI) was calculated using the CalcuSyn software. CI of less than 1 indicates synergism, CI equal to 1 indicates additivity, and CI of greater than 1 indicates antagonism of the two drugs in combination.

	Combination index (CI)								
	786-O	Caki-2	769-P	A704	ACHN	SW839	UM-RC-2	Caki-1	A498
C120	0.83 ± 0.05	0.92 ± 0.07	0.66 ± 0.03	0.81 ± 0.05	0.79 ± 0.06	0.65 ± 0.01	0.85 ± 0.05	1.34 ± 0.09	1.65 ± 0.11
C150	0.67 ± 0.06	0.83 ± 0.04	0.62 ± 0.06	0.54 ± 0.03	0.63 ± 0.04	0.46 ± 0.05	0.76 ± 0.04	1.87 ± 0.12	1.92 ± 0.13
C180	0.58 ± 0.04	0.45 ± 0.03	0.54 ± 0.04	0.45 ± 0.05	0.42 ± 0.02	0.45 ± 0.09	0.69 ± 0.03	1.65 ± 0.13	1.76 ± 0.14

**Table 2:** Combination of ribociclib and IFN-*α* is synergistic in inhibiting the proliferation of some RCC cell lines.

	Combination index (CI)								
	786-0	Caki-2	769-P	A704	ACHN	SW839	UM-RC-2	Caki-1	A489
C120	0.78 ± 0.06	0.78 ± 0.05	0.89 ± 0.06	0.88 ± 0.06	0.65 ± 0.02	0.86 ± 0.05	0.75 ± 0.06	1.65 ± 0.12	1.43 ± 0.18
C150	0.7 ± 0.05	0.65 ± 0.05	0.52 ± 0.04	0.76 ± 0.07	0.43 ± 0.05	0.75 ± 0.03	0.67 ± 0.03	1.56 ± 0.16	1.87 ± 0.1
C180	0.63 ± 0.06	0.53 ± 0.04	0.44 ± 0.03	0.68 ± 0.03	0.35 ± 0.04	0.66 ± 0.04	0.61 ± 0.06	1.45 ± 0.15	1.97 ± 0.23

## Data Availability

All data are contained in the manuscript.

## Supplementary Materials

Supplementary Materials Supplementary Table 1: IC_50_ of the drug alone or in combination in RCC cell lines. Supplementary Fig. 1: ribociclib significantly inhibits the proliferation of 786-O and CaKi-2 cells. Ribociclib at 0.0125 to 0.8 *μ*M was tested. Supplementary Fig. 2: the inhibition curve with the full concentration range of ribociclib on a panel of RCC cell lines. Supplementary Fig. 3: flow cytometry dot plots showing the percentage of Annexin V and 7-AAD staining in RCC cells. Each image was divided into four quadrants: A1, A2, A3, and A4. A1 is Annexin V(+)/7-AAD(-), A2 is Annexin V(+)/7-AAD(+), A3 is Annexin V(-)/7-AAD(-), and A4 is Annexin V(-)/7-AAD(+). The Annexin V(+) population (A1 and A2) was considered apoptotic cells. Supplementary Fig. 4: western blot of p16INK4a in a panel of RCC cell lines showing the baseline level of p16INK4a. Supplementary Fig. 5: western blot of FOXM1, CCNE1, and MSH6 in sensitive and resistant RCC cell lines after ribociclib treatment. Supplementary Fig. 6: mouse body weight in different drug treatment groups.
