# Supplementary material for: Inhibition of the CDK4/6-Cyclin D-Rb Pathway by Ribociclib Augments Chemotherapy and Immunotherapy in Renal Cell Carcinoma
Source: Biomed Res Int. 2020 Jun 11;2020:9525207. doi: 10.1155/2020/9525207 (PMC7306082; doi:10.1155/2020/9525207)
Supplement: Supplementary Materials — Supplementary Table 1: IC50 of the drug alone or in combination in RCC cell lines. Supplementary Fig. 1: ribociclib significantly inhibits the proliferation of 786-O and CaKi-2 cells. Ribociclib at 0.0125 to 0.8 μM was tested. Supplementary Fig. 2: the inhibition curve with the full concentration range of ribociclib on a panel of RCC cell lines. Supplementary Fig. 3: flow cytometry dot plots showing the percentage of Annexin V and 7-AAD staining in RCC cells. Each image was divided into four quadrants: A1, A2, A3, and A4. A1 is Annexin V(+)/7-AAD(-), A2 is Annexin V(+)/7-AAD(+), A3 is Annexin V(-)/7-AAD(-), and A4 is Annexin V(-)/7-AAD(+). The Annexin V(+) population (A1 and A2) was considered apoptotic cells. Supplementary Fig. 4: western blot of p16INK4a in a panel of RCC cell lines showing the baseline level of p16INK4a. Supplementary Fig. 5: western blot of FOXM1, CCNE1, and MSH6 in sensitive and resistant RCC cell lines after ribociclib treatment. Supplementary Fig. 6: mouse body weight in different drug treatment groups. [file 9525207.f1.doc]

**Inhibition of CDK4/6-Cyclin D-Rb pathway by ribociclib augments chemotherapy and immunotherapy in renal cell carcinoma**


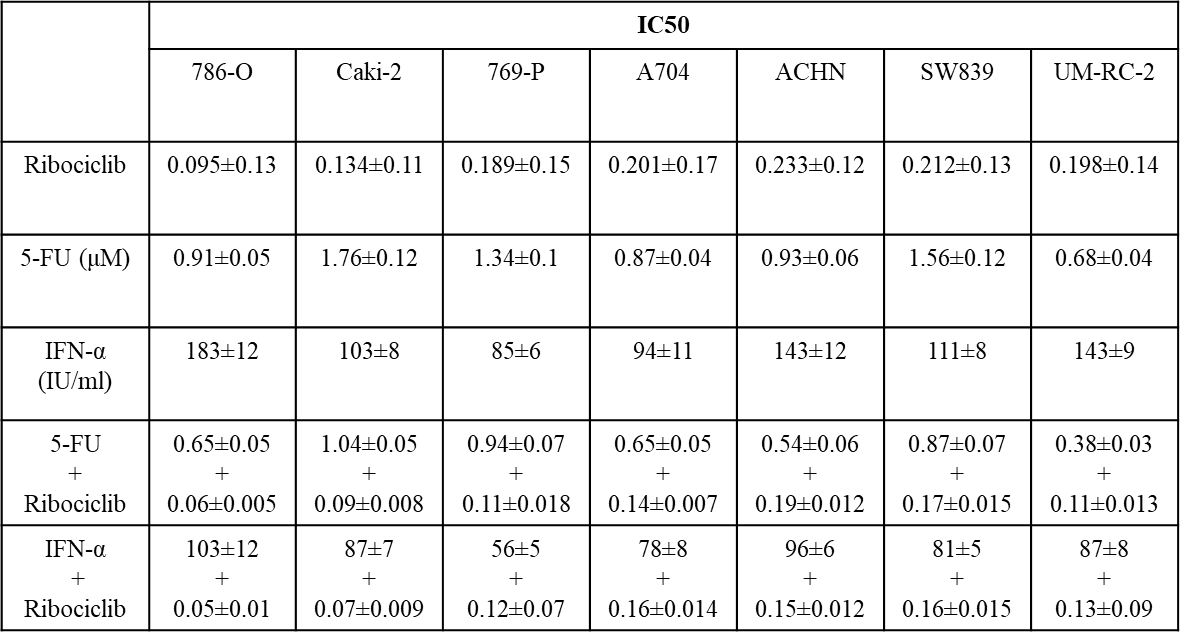


**Supplementary Table 1: IC50 of single drug and combination in RCC cell lines.**

**Supplementary Fig. 1: Ribociclib significantly inhibits proliferation of 786-O and CaKi-2 cells.** Ribociclib at 0.0125 to 0.8 μM were tested.

**Supplementary Fig. 2: The inhibition curve with the full concentration range of ribociclib on a panel of RCC cell lines.**

**Supplementary Fig. 3: Flow cytometry dot plots showing the percentage of Annexin V and 7-AAD staining in RCC cells.** Each image was divided into four quadrants: A1, A2, A3 and A4. A1 is Annexin V (+)/7-AAD(-); A2 is Annexin V(+)/7-AAD(+); A3 is Annexin V (-)/7-AAD(-); A4 is Annexin V (-)/7-AAD(+). The Annexin V (+) population (A1 and A2) were considered as apoptotic cells.

**Supplementary Fig. 4: Western blot of p16INK4a in a panel of RCC cell lines showing the baseline level of p16INK4a.**

**Supplementary Fig. 5: Western blot of FOXM1, CCNE1 and MSH6 in sensitive and resistant RCC cell lines after ribociclib treatment.**

**Supplementary Fig. 6: Mice body weight in different drug treatment groups.**
